# A case report of mania with abnormal cerebral blood flow and cognitive impairment 24 years after head trauma

**DOI:** 10.1186/s12991-020-00282-7

**Published:** 2020-05-12

**Authors:** Hiroki Yoshino, Chieko Aoki, Soichiro Kitamura, Kazuhiko Yamamuro, Shohei Tanaka, Toshifumi Kishimoto

**Affiliations:** Department of Psychiatry, Nara Medical University, 840 Shijocho Kashihara, Nara, 634-8522 Japan

**Keywords:** Manic state, Brain injuries, traumatic, Orbitofrontal cortex, Posterior cingulate cortex

## Abstract

**Background:**

Mania usually occurs secondary to organic etiologies such as head trauma within a short time of the primary condition’s onset; however, there have been a few cases reported in the literature of long time spans before the manifestation of mania. The orbitofrontal cortex has been reported to be associated with manic states in bipolar disorder and with mania-inducing lesions. Head trauma commonly disrupts various cognitive functions, including attention and information processing. Traumatic brain injury patients have been shown to have greater posterior cingulate cortex and precuneus functional connectivity to the rest of the default mode network. We describe a case of secondary mania after head trauma 24 years ago with low blood flow in the orbitofrontal cortex, high blood flow in the posterior cingulate cortex, and impaired cognitive functioning, including impaired attention and lowered processing speed.

**Case presentation:**

We describe a 30-year-old Japanese man with secondary mania and a medical history of head trauma 24 years ago. After head trauma at 6 years of age, the patient first showed apathy as a sign of frontal lobe impairment. After recovering, he experienced no psychiatric problems during adolescence, although he did show disinhibited behavior. At the onset of mania, low blood flow in the OFC and high blood flow in the PCC were observed as well as impaired cognitive function, including inattention and lowered processing speed. Abnormal cerebral blood flow was less prominent and cognitive dysfunction was partially recovered following recovery from mania, but his processing speed remained low.

**Conclusions:**

Although functional recovery from head trauma in childhood is better than that in adulthood, the brain may remain vulnerable for a long time. The risk of psychotic symptoms such as mania should be considered, even if sufficient superficial brain functional recovery is shown.

## Background

Mania is a commonly observed phase of bipolar disorder which can also occur secondary to organic etiologies such as brain ischemia, neurodegenerative disorders, brain tumors, brain infection, and brain trauma. Although secondary mania usually occurs within a short time of the primary condition’s onset, there have been a few cases reported in the literature of long time spans before the manifestation of mania [5]. Generally, functional recovery from head trauma is better in children than in adults. However, even if the recovery in a child seems sufficient to allow for a normal future, the brain may remain vulnerable to stress for many years. This vulnerability can eventually result in the development of a mental disorder such as mania.

Both decreased orbitofrontal cortex (OFC) activity during rest [1] and an underactive inferior frontal cortex [2] have been reported to be associated with manic states in bipolar disorder. Furthermore, networks that include the OFC, the dorsolateral prefrontal cortex, and the temporal lobe are selectively disrupted in mania-inducing lesions [3], further confirming the importance of the OFC in manic pathophysiology.

Head trauma commonly leads to diffuse axonal injury, which disrupts cognitive functions such as attention and information processing. Traumatic brain injury patients have been shown to have greater posterior cingulate cortex (PCC) and precuneus functional connectivity to the rest of the default mode network, and patients with greater functional connectivity within these regions show less cognitive impairment [4]. The default mode network, including the PCC, might be activated to compensate for neural circuit dysfunction from head trauma.

We describe a case of secondary mania after head trauma 24 years ago with low blood flow in the OFC, high blood flow in the PCC, and impaired cognitive functioning, including impaired attention and lowered processing speed.

### Case presentation

A 30-year-old Japanese man with no personal or familial psychiatric history was admitted to a psychiatric hospital for manic episode. His medical history included head trauma by traffic accident at the age of 6 years. He experienced severe impaired consciousness for 2 weeks after the head trauma. After discharge he showed a flat affect, which resolved after 3 months. Thereafter, he showed an inability to control his emotions for some time, but this eventually resolved, and he lived a stable life without major problems.

At the age of 12 years, the patient passed an entertainment office audition and subsequently performed on TV programs and on the stage. During high school, he studied in Australia and obtained tattoos on his whole back, which is uncommon behavior for a Japanese student. During college, he was the captain of a dance team. At 21 years, he had a child. Thus, his behaviors from the ages of 12 to 21 could be interpreted as showing a disinhibited tendency. After college graduation, he initially worked on a motor race rescue team for 4 years before switching to work in his family’s company. At 29 years, he experienced a problem with his relationship with his sister and became estranged from his family.

The patient began working as an apparel retailer and was taking lessons for Japanese classical dance and classical ballet. He frequently went dancing in nightclubs, reversing his day/night cycle. According to the patient’s wife, the patient stated, “I communicate with the universe” during this period. In June of 2016, at the age of 30, he was scouted in a night club by a record company, and he ended up paying the company several thousand dollars to produce his music video. In addition, his schedule grew busier because of training in both Noh theater (Noh is a form of traditional Japanese dance-drama) and ballet. In August, after seeing a Noh performance, he showed symptoms of megalomania by claiming “Because Noh is a world heritage, I would not be guilty even if I killed a man.” and “The world goes around me.” At his grandmother’s funeral at the end of September, he stated, “I worship nature and attend as God”. After the funeral, he suddenly went to a shrine and prayed to cleanse his unclean body. When he returned home, he quarreled with his mother, yelling and behaving incoherently.

**Fig. 1:**
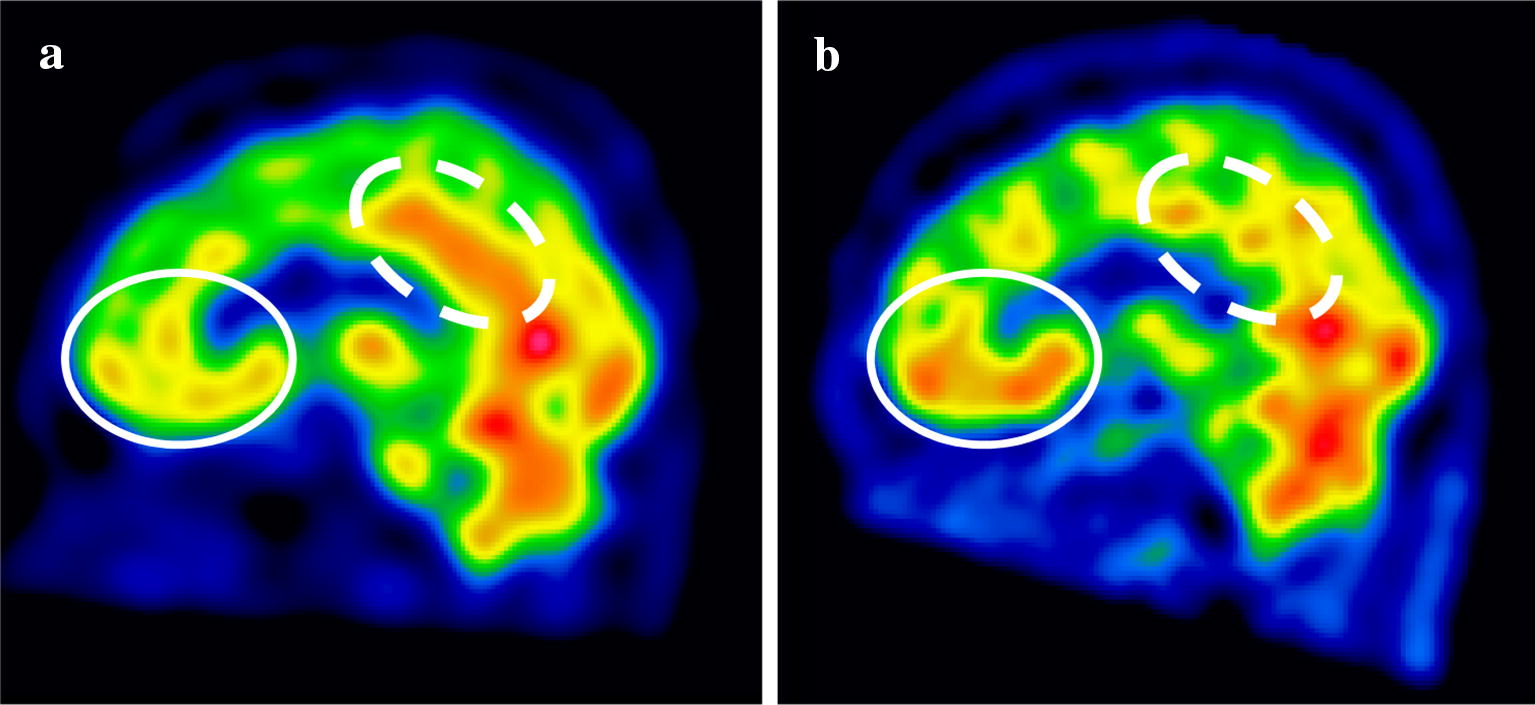
Cerebral blood flow imaged by SPECT during mania and after recovery. **a** During mania, the blood flow in the orbitofrontal cortex (OFC) was low (solid-line circle), while the blood flow in the posterior cingulate cortex (PCC) was high (dashed circle). **b** After recovery, blood flow was normalized in both the OFC and the PCC

On October 1, he dashed naked out of the house and repeatedly made emergency calls to police to ask, “Who am I?” He also brazenly took many books out of a bookstore without paying. Because of these incoherent behaviors, he was voluntarily admitted to a hospital. Although pharmacological treatment with aripiprazole was initially adopted, his symptoms continued to deteriorate. The patient purchased an expensive Japanese sword and telescope via a telephone service and displayed sexually disinhibited behavior in the hospital. The admission was changed from voluntary to involuntary. On November 8, the patient was referred to our hospital to have a more detailed examination. On admission to our facility, the patient was apathic rather than hyperactive. Electroencephalography and brain computerized tomography (CT) revealed no notable results. Magnetic resonance imaging was not available due to his tattooed back, but single photon emission computed tomography (SPECT) revealed low blood flow in the OFC and high blood flow in the PCC (Fig. 1a). According to the Wechsler Adult Intelligence Scale–Third (WAIS-III), the patient’s total IQ was 85 (verbal IQ: 90, performance IQ: 82). In particular, his WAIS-III scores for letter–number sequencing, picture completion, digit symbol-coding, and symbol research were low. He scored an 81/85 (low average) on the Behavioral Assessment of the Dysexecutive Syndrome (BADS), and his score on the Wisconsin Card Sorting Test (WCST) was normal. His performance on the trail-making test was accurate, but he processed slowly. Together, these tests revealed that the patient had a slow processing speed and lowered attention (Table 1).

**Table 1:** Neuropsychological test results during mania and after recovery

			Manic state	After recovery	Normal
WAISIII	Total IQ		85	96	
	Verbal IQ		90	97	
		Verbal comprehension index	84	93	
		Vocabulary	7	8	
		Similarities	9	12	
		Information	5	6	
		Comprehension	11	10	
		Working memory index	85	96	
		Arithmetic	11	12	
		Digit span	7	9	
		Letter–number sequencing	5	7	
	Performance IQ		82	95	
		Perceptual organization index	91	99	
		Picture completion	6	8	
		Block design	11	11	
		Matrix reasoning	9	11	
		Processing speed index	72	81	
		Digit symbol-coding	4	6	
		Symbol research	6	7	
BADS		Rule shift cards test	3	4	3.56
		Action program test	4	4	3.77
		Key search test	2	2	2.6
		Temporal judgment test	1	1	2.15
		Zoo map test	2	3	2.44
		Six elements test	3	4	3.52
		Total score	15	18	18.05
		Standard	85	100	
		Standard by age	81	97	
WCST		Categories achieved	6	5	5.3
		Total errors	12	13	11.2
		Perseverative errors of Milner	0	0	3.1
		Perseverative errors of Nelson	1	1	1.6
		Maximum classification score	2	5	6.6
		Difficulty of maintaining set	0	0	0.8
		Unique errors	0	0	0
Trail-making test		Test A	124 s	98 s	70.9 s
		Test B	105 s	105 s	90.1 s

During mania, WAIS-III, BADS, and trail-making test scores were low, but these were improved after recovery. WCST score was not affected during mania. WAIS-III scores associated with attention, i.e., digit span, letter–number sequencing, and picture completion, were low during mania and improved after recovery. WAIS-III scores associated with processing speed, i.e., digit symbol-coding and symbol research, were low during mania and partially improved after recovery

After admission to our hospital, the patient was treated with carbamazepine in addition to aripiprazole. He gradually became coherent enough to claim that he did not have any fantastical feelings, and finally his megalomania and irritability disappeared. 9 months after discharge, in February 2017, he reported that his life had returned to how it was before the onset of mania. Reflective of this recovery, he showed a WAIS-III total IQ of 96 (verbal IQ: 97, performance IQ: 95). Other tests further confirmed his improvement, although he still showed a low processing speed and low attention (Table 1). Hypo-activity in the OFC and hyper-activity in the PCC were no longer detected by SPECT (Fig. 1b).

### Discussion and conclusions

We encountered a case of secondary mania with a head trauma 24 years ago. After head trauma at 6 years of age, the patient first showed apathy as a sign of frontal lobe impairment. After recovering, he experienced no psychiatric problems during adolescence, although he did show disinhibited behavior. At the onset of mania, low blood flow in the OFC and high blood flow in the PCC were observed as well as impaired cognitive function, including inattention and lowered processing speed. Abnormal cerebral blood flow was less prominent and cognitive dysfunction was partially recovered following recovery from mania, but his processing speed remained low.

It has been reported that mania induced by head trauma in adulthood usually occurs relatively soon after the injury [5]. Generally, the functional recovery from head trauma in childhood is better than that in adulthood. The current case patient experienced head trauma in childhood, after which he superficially showed sufficient brain function recovery to allow him to live a stable life for 24 years without any major problems. However, his brain may have remained vulnerable, and he showed a tendency of disinhibition and low processing speed. It is possible that the patient being overloaded with stress caused a manic state and caused compensated dysfunction arising from head trauma to become evident.

Although no visual morphological abnormalities were observed by CT at the onset of mania, head trauma in early childhood may have conferred vulnerability on the OFC, which may in turn have led to the tendency toward disinhibited behaviors during adolescence. Under stress, vulnerability of the OFC might have led to lowered cerebral blood flow and subsequent mania [3]. OFC dysfunction could also explain the patient’s apathic behavior when he was referred to our hospital. Head trauma commonly disrupts brain networks following diffuse axonal injury, which causes lowered processing speed and inattention, and increased PCC activity within the default mode network can act as an adaptive response [4]. The case patient might have had hyper-activity of the PCC prior to the onset of mania, and during mania this might have become evident as increased blood flow in the PCC. Low processing speed and inattention during and after mania may have been due to neuronal network dysfunction caused by diffuse axonal injury from the head trauma [4]. Because the patient’s brain might show potential vulnerability in the future, the risk of mania should be lowered using pharmacotherapy and stress management.

## Data Availability

The datasets used and/or analyzed during the current case report are available from the corresponding author on reasonable request.

## Abbreviations

OFC: Orbitofrontal cortex
PCC: Posterior cingulate cortex
CT: Computerized tomography
SPECT: Single photon emission computed tomography
WAIS-III: The Wechsler Adult Intelligence Scale–Third
